# Cosmetic Gynecology: Is There Enough Exposure in the Obstetrics and Gynecology Residency?

**DOI:** 10.7759/cureus.65564

**Published:** 2024-07-28

**Authors:** Annika Sinha, Meng Yao, Sunny Lee, Nicole Wood, Vicki Reed, Shannon L Wallace

**Affiliations:** 1 Urogynecology, Duke University, Durham, USA; 2 Quantitative Health Sciences, Cleveland Clinic Foundation, Cleveland, USA; 3 Obstetrics and Gynecology, Cleveland Clinic Foundation, Cleveland, USA; 4 Urogynecology, Hartford Hospital, Hartford, USA; 5 Urogynecology, Cleveland Clinic Foundation, Cleveland, USA

**Keywords:** surgical-education, general gynecology, obstetrics and gynecology residency, medical resident education, cosmetic gynecology

## Abstract

Introduction

While cosmetic gynecology has gained popularity, the training experience for obstetrics and gynecology residents in this area is limited and not standardized. The primary objective was to investigate the exposure of obstetrics and gynecology residents to cosmetic gynecologic procedures including hymenoplasty, labia majora reduction, vaginoplasty, G-spot amplification, labiaplasty, clitoral hood reduction, and vaginal laser therapy. The secondary objective was to assess their comfort in performing the procedures after graduation.

Methods

This is a non-validated cross-sectional survey of obstetrics and gynecology residents and their exposure to cosmetic gynecology procedures. Using the Fellowship and Residency Electronic Interactive Database Access System, the Accreditation Council for Graduate Medical Education-accredited Obstetrics and Gynecology residency programs in the United States were identified, and the residency program coordinators were asked to distribute the survey. Data regarding demographics, program-specific characteristics, and exposure to certain cosmetic gynecology procedures were obtained and reviewed. Participants’ reported comfort with the independent practice of certain cosmetic gynecology procedures after graduation was also obtained. Descriptive statistics were completed.

Results

A total of 96 responses were received. Approximately 50% of participants were exposed to cosmetic gynecology during training. Moreover, 70.9% of residents disagreed that they would feel confident in defining the included procedures. Furthermore, 87.5% disagreed that they would feel comfortable performing the procedures independently. A minority of participants were also unsure of the indication for cosmetic gynecology procedures, with 15.4%, 7.1%, 5.7%, and 4% unaware of the surgical indication for hymenoplasty, vaginoplasty, labiaplasty, and labia majora reduction, respectively. No participant knew the indication of surgery for vaginal laser therapy or clitoral hood reduction.

Conclusions

In the setting of current cosmetic gynecology training, nearly 90% of residents were not comfortable with these surgeries after graduation. Exposure to cosmetic gynecology for obstetrics and gynecology residents was limited. Without adequate exposure, residents may face difficulty performing procedures and managing complications after graduation. Therefore, standardizing resident training for cosmetic gynecology should be considered.

## Introduction

Cosmetic gynecology is a fast-growing subspecialty within obstetrics and gynecology. With the advent of social media and the focus on aesthetic outcomes, patients are increasingly interested in cosmetic procedures, including hymenoplasty, labia majora reduction, vaginoplasty, G-spot amplification, labiaplasty, clitoral hood reduction, and other genital enhancements [1]. In the United States, 10,817 labiaplasty procedures were completed in 2017, which represents a 217% increase from the five years prior [1]. Currently, the most common cosmetic gynecologic procedure is labiaplasty [2]. Despite the profound interest and growth in this field, there is a lack of high-quality evidence on long-term outcomes after these procedures.

According to publication data, publishing providers of cosmetic gynecology procedures include general obstetrics and gynecology physicians, plastic surgery-trained physicians, subspecialists within obstetrics and gynecology, oral-maxillofacial surgeons, non-physician providers, and non-licensed providers without medical training [1]. Initially, the role of gynecologists in cosmetic gynecology was unclear [3-5]. In 2007, the American College of Obstetricians and Gynecologists (ACOG) advised against performing cosmetic procedures due to a lack of safety and efficacy data [3]. In 2012, Iglesia encouraged patients and providers to embrace anatomic differences and avoid medicalization of normal characteristics in an editorial in Obstetrics and Gynecology [4]. Similarly, Rogers published an editorial in the American Journal of Obstetrics and Gynecology that warned that those undergoing labiaplasty often had normal anatomy [5]. However, as the field has evolved, a focus on supporting patient autonomy and providing safe care has emerged. In a 2014 editorial in the American Journal of Obstetrics and Gynecology, Pauls advocated for the obstetrician and gynecologist’s role in cosmetic gynecology, stating that “we are the correct physicians to treat women requesting labiaplasty” [6]. In practice, aesthetic providers, primarily with plastic surgery training, provide these procedures, and most cosmetic gynecology education stems from post-residency training through independent cosmetic surgical courses or cosmetic fellowships.

After this shift, the ACOG revised its stance on cosmetic gynecology. In the recently affirmed Practice Guideline #795, ACOG recommends that all obstetrician-gynecologists providing cosmetic gynecology should have sufficient training [2]. Additionally, providers should recognize sexual function disorders, depression, anxiety, body dysmorphia, and other conditions prior to offering cosmetic gynecologic procedures [2]. It is also recommended that physicians reassure patients that the appearance of external genitalia can vary and is altered by aging, childbirth, and other life experiences. Finally, those providing cosmetic gynecology should be transparent about their outcomes and experiences, given the overall limited data on this topic. Patients should be counseled about complications including bleeding, pain, scarring, the need for reoperation, and disapproval of the aesthetic outcome.

Despite the tremendous growth of cosmetic gynecology, resident training is not standardized, and resident experience and exposure likely differ among training programs. The Accreditation Council for Graduate Medical Education (ACGME) does not currently require any cosmetic gynecology procedures as part of the minimum case requirements for graduation [7].

The primary objective of this study was to investigate the exposure of current obstetrics and gynecology residents to cosmetic gynecologic procedures, including hymenoplasty, labia majora reduction, vaginoplasty, G-spot amplification, labiaplasty, clitoral hood reduction, and vaginal laser therapy. The secondary objective was to assess their confidence in their knowledge about the procedures and their comfort in performing the procedures after graduation.

The findings of this study were presented as an electronic poster at the Society of Gynecologic Surgeons 49th Annual Scientific Meeting in Tucson, Arizona, United States, from March 19 to 22, 2023.

## Materials and methods

This cross-sectional survey-based study of active obstetrics and gynecology residents from graduating class years 2023-2027 in the United States was conducted at the Cleveland Clinic Foundation, Cleveland, United States. A 25-item non-validated anonymous English-language questionnaire regarding resident exposure to cosmetic gynecology, resident comfort in defining and completing cosmetic gynecology procedures, and resident ability to diagnose concurrent psychiatric conditions was created in REDCap and was electronically distributed (Appendix 1). The survey was structured into four main sections: (1) demographics and residency program characteristics; (2) exposure to certain procedures (hymenoplasty, labia majora reduction, vaginoplasty, G-spot amplification, labiaplasty, and clitoral hood reduction); (3) preoperative evaluation and counseling; and (4) comfort with future procedures. In the sections on preoperative knowledge (3) and comfort with future procedures (4), answers were graded on ordinal scales with the following levels: strongly disagree, somewhat disagree, neither agree nor disagree, somewhat agree, or strongly agree.

Using the Fellowship and Residency Electronic Interactive Database Access (FREIDA) System, the ACGME-accredited obstetrics and gynecology residency programs in the United States were identified, and the residency program coordinators were contacted. The survey was emailed to the program coordinators, who served as a proxy for the primary contact for each of the 295 ACGME-accredited residencies in all 50 states of the United States. Each program coordinator was asked to forward the survey to their current residents (2019-2023). Responses were collected between April 2023 and June 2023 in order to obtain data from the end of the graduate year to maximize the potential of exposure to cosmetic gynecology procedures during that respective year.

The residents ranged from postgraduate year 1 to postgraduate year 4. Participation was voluntary, and no compensation was given. We excluded residents from training programs where the residency program coordinator’s email information was not available on FREIDA. Approval was obtained by the Institutional Review Board (IRB 23-343).

Statistical analysis

Descriptive statistics were used to analyze the survey data. Pearson’s chi-squared test and Fisher’s exact test were used to compare resident exposure to specific cosmetic gynecology procedures by postgraduate year of training and to assess resident understanding of the indication of surgery for specific cosmetic gynecology procedures. The Kruskal-Wallis test was used to compare resident comfort with diagnosing concurrent psychiatric conditions and to compare resident comfort with defining and performing cosmetic gynecology procedures. Statistical significance was determined by p < 0.05. The SAS statistical package (SAS Institute, Cary, North Carolina, United States) was used for statistical analysis.

## Results

Demographics

A total of 96 responses from US obstetrics and gynecology residents were collected. Most participants were cis-gendered females (89.6%) and White (61.5%). A total of 90.6% of participants were not of Latinx, Hispanic, or Spanish origin. Moreover, 42.7% of participants were located primarily in the Midwest (Ohio, Indiana, Michigan, Illinois, Missouri, Wisconsin, Minnesota, Iowa, Kansas, Nebraska, South Dakota, and North Dakota), 36.5% were located in the Northeast (Connecticut, Maine, Massachusetts, New Hampshire, Rhode Island, New Jersey, New York, Vermont, Delaware, Maryland, and Pennsylvania), 10.4% in the Southwest (Texas, Oklahoma, New Mexico, Arizona), 7.3% in the Southeast (West Virginia, Kentucky, Tennessee, North Carolina, South Carolina, Georgia, Alabama, Mississippi, Arkansas, Louisiana, and Florida), and 3.1% in the West (Colorado, Wyoming, Montana, Idaho, Washington, Oregon, Utah, Nevada, California, Alaska, and Hawaii). Most participants (58.3%) were training at university-based residency programs, followed by 34.4% at community-based university-affiliated programs, 8.3% at community-based programs, and 0% at military-based programs. When asked for the most common insurance coverage for their patients, 64.6% reported that most of their patients used Medicaid insurance; 19.8% reported that most of their patients used private insurance; 5.2% reported that most of their patients used Medicare; and 10.4% of participants were uncertain about the predominant insurance coverage for their patients. Results are shown in Table 1.

**Table 1:** Demographic data of obstetrics and gynecology residents who participated in the study from April to June 2023

Category	N (%)
Level of training	
PGY-1	22 (23%)
PGY-2	27 (28%)
PGY-3	18 (19%)
PGY-4	29 (30%)
Gender	
Cis-gendered male	10 (10%)
Cis-gendered female	86 (89%)
Trans-gendered male	0 (0%)
Trans-gendered female	0 (0%)
Non-binary	0 (0%)
Other	0 (0%)
Prefer not to answer	0 (0%)
Race	
Black	15 (16%)
Asian	14 (15%)
White	59 (62%)
Native Hawaiian or other Pacific Islander	0 (0%)
Other	7 (7%)
Prefer not to answer	1 (1%)
Ethnicity	
Hispanic, Latinx, or Spanish origin	9 (9%)
Region	
Northeast	35 (37%)
Southeast	7 (7%)
Midwest	41 (43%)
Southwest	10 (10%)
West	3 (3%)
Type of program	
Community-based	8 (8%)
Community-based university-affiliated	33 (34%)
University-based	56 (58%)
Military-affiliated	0 (0%)
Type of insurance	
Medicaid	62 (65%)
Medicare	5 (5%)
Private	19 (20%)
Uncertain/unsure	10 (10%)

Exposure to specific cosmetic gynecology procedures

**Figure 1:**
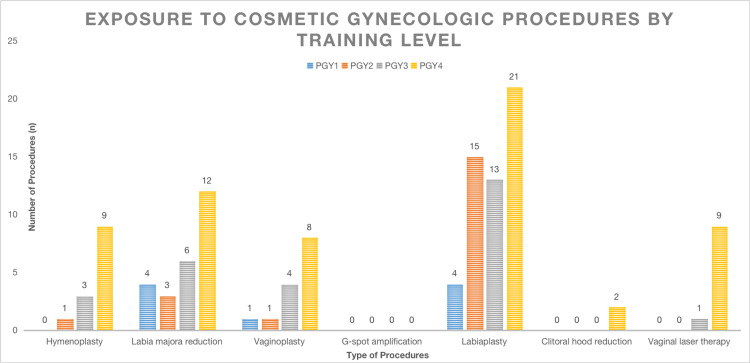
Surgical exposure to specific cosmetic gynecology procedures, categorized by participant training level

Nearly 50% of participants reported exposure to faculty who completed cosmetic gynecology procedures during their training experience. The most common procedure that residents were exposed to was labiaplasty (55.2%), followed by labia majora reduction (26.3%), vaginoplasty (14.6%), hymenoplasty (13.5%), vaginal laser therapy (10.4%), clitoral hood reduction (2.1%), and G-spot amplification (0%). Exposure to all procedures, except G-spot amplification and clitoral hood reduction, trended toward increased exposure with increasing training level (Figure 1).

Preoperative evaluation and counseling

**Figure 2:**
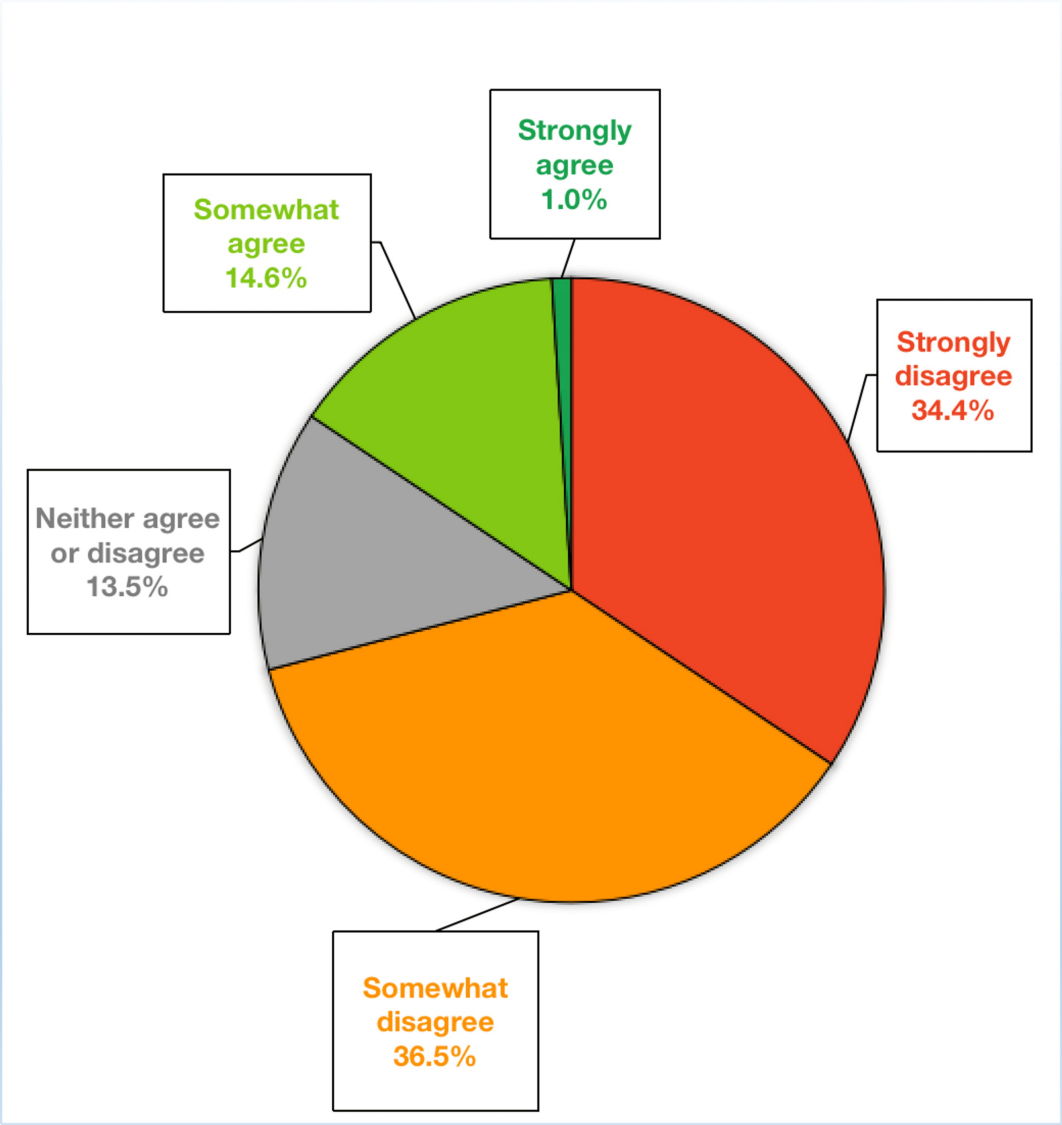
Participants’ responses to the survey item “As an OB/GYN resident, I can confidently define all the procedures,” which include hymenoplasty, labia majora reduction, vaginoplasty, G-spot amplification, labiaplasty, and clitoral hood reduction

We asked a series of non-validated questions regarding resident knowledge about cosmetic gynecology procedures and the diagnosis of concurrent psychosocial conditions in these patients. When asked, “I can confidently define all of these procedures: hymenoplasty, labia majora reduction, vaginoplasty, G-spot amplification, labiaplasty, and clitoral hood reduction,” most participants (70.9%) disagree, and 34.4% of participants strongly disagree (Figure 2).

More than half of the participants felt comfortable diagnosing depression, anxiety, and body dysmorphia in patients seeking cosmetic gynecologic procedures. The participants were asked if they knew or were unsure about the preoperative indications for cosmetic procedures. A total of 15.4% of participants were unaware of the indication for surgery in the setting of hymenoplasty, and 7.1% of participants were unaware of the indication for surgery in the setting of vaginoplasty. Similarly, 5.7% and 4.0% were unaware of the surgical indication for labiaplasty and labia majora reduction, respectively. Of those who were involved in these procedures, no participant knew the indication of surgery for vaginal laser therapy or clitoral hood reduction.

Comfort with future procedures

**Figure 3:**
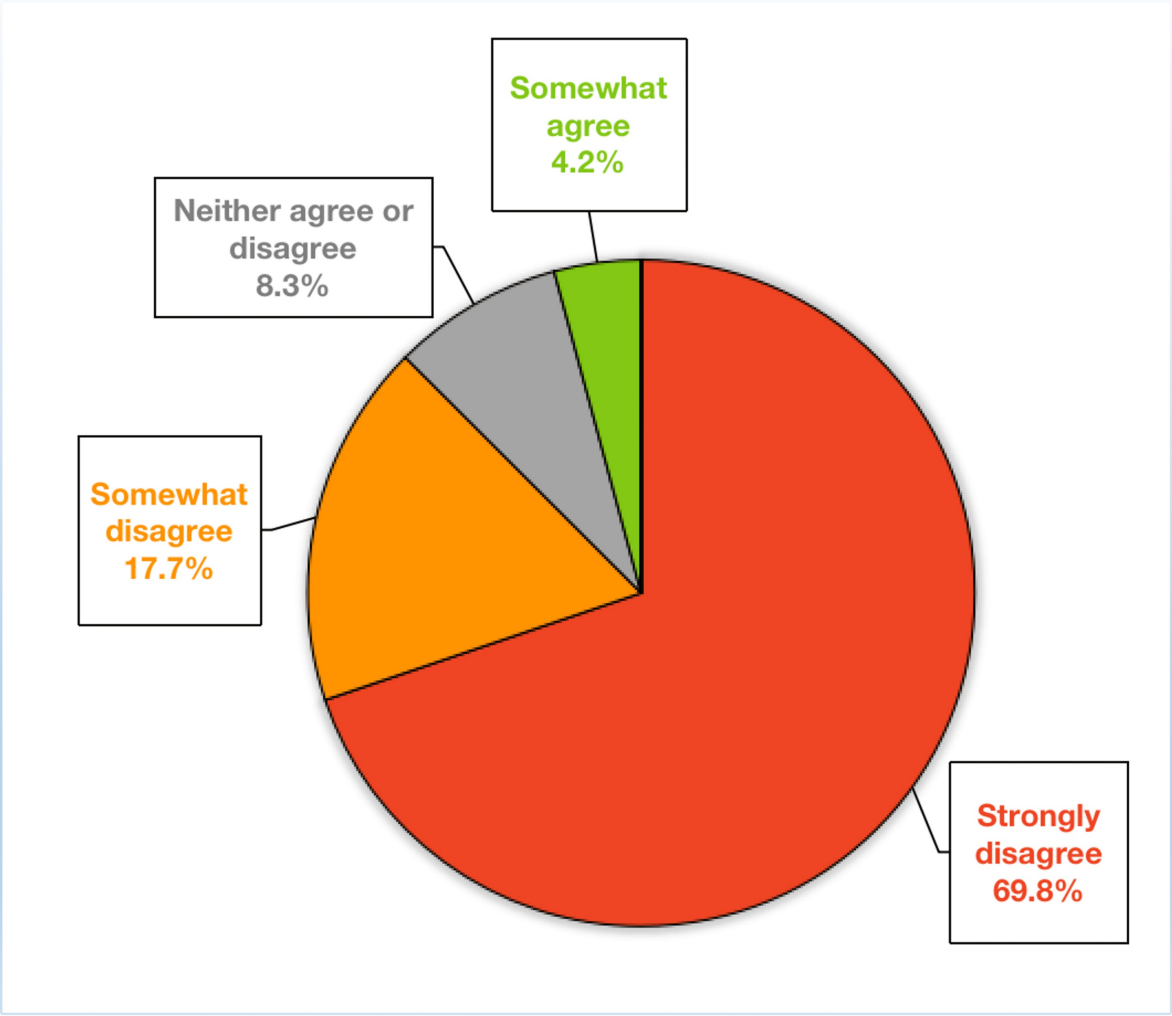
Participants’ responses to the survey item “Based on my training, I will feel comfortable performing cosmetic gynecology procedures independently after graduation,” which include hymenoplasty, labia majora reduction, vaginoplasty, G-spot amplification, labiaplasty, and clitoral hood reduction

Participants were asked their opinion on the following statement: “Based on my training, I will feel comfortable performing cosmetic gynecology procedures independently after graduation.” Nearly nine out of 10 residents (87.5%) disagreed with this statement, and 69.8% strongly disagreed. Only 4.2% of participants somewhat agreed with the above statement, and 0% of all participants strongly agreed that they would feel comfortable with performing cosmetic gynecology procedures independently after graduation (Figure 3).

## Discussion

Principal findings

In this small-sized study, approximately one in two OB/GYN residents had exposure to faculty completing cosmetic gynecology surgeries. Only 55% of residents were exposed to the most commonly performed procedure (labiaplasty). About half of the participants felt comfortable diagnosing psychiatric conditions that may present in patients expressing desires for cosmetic gynecology procedures. A total of 70.9% of residents cannot confidently define specific cosmetic gynecology procedures, and no participant in the study reported that they would strongly agree with the statement: “Based on my training, I will feel comfortable performing cosmetic gynecology procedures independently after graduation.”

Results in the context of published literature

Our study highlights the imbalance between demand in cosmetic gynecology and resident training education, as there are no previously published studies or guidelines on formalized resident training in cosmetic gynecology. The ACOG recommends that “obstetricians-gynecologists who perform cosmetic procedures should be adequately trained, experienced, and clinically competent to perform the procedure” [2]. However, the ACGME OB/GYN residency training minimum requirements do not include any minimums for cosmetic gynecology procedures [7]. Therefore, resident education in cosmetic gynecology procedures likely varies based on the institution and shapes future practice patterns in cosmetic gynecology.

Clinical implications

Data from the last 10 years shows an increased demand for cosmetic gynecology in the United States as well as internationally [1]. In India, there was an increase in cosmetic gynecology procedures from 3.9% in 2012 to 28.97% in 2015 [8]. Despite the steady increase in procedure volume, there is not a formalized, widespread curriculum and training protocol for these surgeries. According to the International Urogynecological Association (IUGA), there are more than 20 independent cosmetic gynecologic symposiums and other resources that may lack rigorous review [9]. Given this, IUGA has created a Cosmetic Gynecology Special Interest Group to improve education in cosmetic gynecology. Similarly, the Society of Gynecologic Surgeons and the American Urogynecologic Society (AUGS) have both actively supported education in cosmetic gynecology. IUGA and AUGS have published a standardized joint terminology guideline in cosmetic gynecology [10]. It is important to note that as acceptance for performing cosmetic gynecology procedures has increased, some forms of education, primarily short interval, often expensive surgical courses, have emerged [11]. Although there has been an increase in preceptor-drive apprenticeships or courses to teach these concepts, this mode of education is not incorporated into the traditional OB/GYN residency programming [12]. Therefore, OB/GYN residents may not be routinely exposed to this education since this programming stems from subspecialty academic societies or preceptor-based courses [13].

Our data show that there is a gap in resident education in cosmetic gynecology with low rates of comfort with independent practice of cosmetic gynecology after graduation in the United States. With nearly 90% of OB/GYN-trained participants reporting some level of discomfort with cosmetic gynecology after graduation, residents may not offer these services; however, they may still be responsible for the management of complications. For those who will practice cosmetic gynecology, the clinical implications of fragmented training may lead to difficulty in patient selection. Improving resident education is not only important for patient safety but also in line with the ACOG’s recommendation that all gynecologists with the potential to provide cosmetic gynecology should be adequately trained.

Future implications

As mentioned, there are currently no published studies on cosmetic gynecology resident education. More comprehensive studies are needed to adequately understand the current diversity in resident education and training related to these procedures. Similarly, studies on the impact of the amount of resident exposure on surgical outcomes are also required to evaluate the effectiveness of methods of resident education. Additionally, evaluating the current practice of early-career OB/GYN physicians in the United States may provide insight into their comfort level with cosmetic gynecology and offer a reflection of their residency experience and education. Similar studies have been completed in Saudi Arabia, revealing that those who practice and those who do not practice cosmetic gynecology both counsel patients and can have positive attitudes toward this type of surgery after experience and education [14]. Finally, especially in the United States, it is important to understand the implications of limited resident education in the context of malpractice. In a review of 64 malpractice cases regarding cosmetic gynecology, most cases occurred in the setting of out-of-scope practice, which is legally defined as practicing outside of current ACGME procedural requirements [15]. As previously mentioned, there are no current ACGME requirements for cosmetic gynecology procedures; therefore, this type of legal definition could significantly impact any future OB/GYN physicians performing cosmetic gynecology procedures. Therefore, future studies on the current landscape of OB/GYN physician training are required to prompt more standardization of such curricula.

Strengths and limitations

Strengths of this study include the evaluation of a learning gap in obstetrics and gynecology training as demand for and popularity of cosmetic gynecology increase. Based on our most recent literature reviews, this is the first study to evaluate the obstetrics and gynecology residents’ experience with cosmetic gynecology training and comfort with providing this type of care in the future. Despite the anonymous survey design, we were able to receive responses from participants in all regions of the United States, with a diversity in self-reported program types. The study was also conducted at the end of the educational year (April to June), so residents were able to evaluate their surgical exposure for their PGY level.

Limitations of this study include a low response rate of 96 participants and a small sample size, which makes it difficult to generalize our findings to the entire US OB/GYN residency cohort. Given that there is limited literature on this clinical topic, it was not possible to complete a true power calculation, and therefore, a descriptive design was chosen. We believe that there are limitations inherent to its descriptive design. Although greater than 50% of the participants were not from the Midwest, there may have been some institutional bias in these results. As our study was an opt-in survey for all residents, we could have introduced selection bias into the design. Additionally, there was homogeneity in the demographic characteristics of participants, with most individuals identifying as cis-gendered, white, non-Hispanic females. The study questions were not validated, as there is no current validated survey for this clinical question. Another limitation is that only a select number of cosmetic gynecology procedures were highlighted, and although participants could select more than one answer for the “indication”-related questions, the survey included a grouping of procedures in questions, which could have biased results. There was no further evaluation or reporting of case numbers to better quantify exposure. Finally, we were unable to verify the receipt and distribution of the study after the initial email to program coordinators. Specific barriers to education were not elucidated in this study and would be important for future studies to design robust surgical curricula. Examples of current resident curricula were also not investigated.

## Conclusions

In this small study of 96 participants, nearly 90% of residents reported feeling uncomfortable providing cosmetic gynecology care independently after graduation. While many obstetrics and gynecology physicians may not offer aesthetic services post-graduation, understanding the surgical principles of cosmetic gynecology procedures could be beneficial for managing complications, in light of ACOG’s current guidelines on cosmetic gynecologic procedures. Despite its limitations in scope and size, this study suggests the potential benefit of standardizing resident education, experience, and exposure to cosmetic gynecology. Implementing such a structure could enhance resident learning and contribute to safer and more comprehensive patient care in cosmetic gynecology.

## Appendices

Appendix 1

**Table 2:** Participant survey For questions 4, 10, 11, 13, 15, 17, 19, 21, and 23, participants were able to select multiple answers.

Survey item	Answer choice
1. What year of OB/GYN residency training are you currently in?	PGY-1
	PGY-2
	PGY-3
	PGY-4
2. Which gender do you identify with the most?	Male
	Female
	Non-binary
	Prefer not to answer
3. Are you of Hispanic, Latino, or Spanish origin?	Yes
	No
	Prefer not to answer
4. How would you describe yourself?	Black or African American
	Asian
	American Indian or Alaska Native
	White
	Native Hawaiian or other Pacific Islander
	Other
	Prefer not to answer
5. What region is your institution located in?	Northeast
	Southeast
	Southwest
	West
6. How is your program classified?	Community-based
	Community-based university-affiliated
	University-based
7. What is the most common insurance that benign gynecologic patients have in your training program (including faculty and resident continuity care patients)?	Medicaid
	Medicare
	Private
	Uncertain
8. Do any faculty members at your institution perform the following cosmetic gynecology procedures, including hymenoplasty, labia majora reduction, vaginoplasty, G-spot amplification, labiaplasty, clitoral hood reduction, and vaginal laser therapy?	Yes
	No
	Uncertain
9. As an OB/GYN resident, I can confidentially define all of these procedures: hymenoplasty, labia majora reduction, vaginoplasty, G-spot amplification, labiaplasty, clitorial hood reduction, and vaginal laser therapy?	Strongly disagree
	Somewhat disagree
	Neither agree nor disagree
	Somewhat agree
	Strongly agree
10. Have you participated in hymenoplasty during your training program?	Yes
	No
	Uncertain
11. If you have participated in hymenoplasty, do you know the indication for the surgery? If you have not seen this procedure, please check not applicable.	Pain
	Sexual dysfunction
	Obstetric-related injuries
	Prolapse procedures
	Gender dysphoria/gender affirmation surgery
	Interference with sports
	Vaginal atrophy
	Reversal of female genital mutilation
	Body dysmorphia and other psychiatric conditions
	Unsure of the indication for surgery
	Not applicable
12. Have you participated in labia majora reduction during your training program?	Yes
	No
	Uncertain
13. If you have participated in labia majora reduction, do you know the indication for the surgery?	Pain
	Sexual dysfunction
	Obstetric-related injuries
	Prolapse procedures
	Gender dysphoria/gender affirmation surgery
	Interference with sports
	Vaginal atrophy
	Reversal of female genital mutilation
	Body dysmorphia and other psychiatric conditions
	Unsure of the indication for surgery
	Not applicable
14. Have you participated in vaginoplasty during your training program?	Yes
	No
	Uncertain
15. If you have participated in vaginoplasty, do you know the indication for the surgery? If you have not seen this procedure, please check not applicable.	Pain
	Sexual dysfunction
	Obstetric-related injuries
	Prolapse procedures
	Gender dysphoria/gender affirmation surgery
	Interference with sports
	Vaginal atrophy
	Reversal of female genital mutilation
	Body dysmorphia and other psychiatric conditions
	Unsure of the indication for surgery
	Not applicable
16. Have you participated in G-spot amplification during your training program?	Yes
	No
	Uncertain
17. If you have participated in G-spot amplification, do you know the indication for the surgery? If you have not seen this procedure, please check not applicable.	Pain
	Sexual dysfunction
	Obstetric-related injuries
	Prolapse procedures
	Gender dysphoria/gender affirmation surgery
	Interference with sports
	Vaginal atrophy
	Reversal of female genital mutilation
	Body dysmorphia and other psychiatric conditions
	Unsure of the indication for surgery
	Not applicable
18. Have you participated in labiaplasty during your training program?	Yes
	No
	Uncertain
19. If you have participated in labiaplasty, do you know the indication for the surgery? If you have not seen this procedure, please check not applicable.	Pain
	Sexual dysfunction
	Obstetric-related injuries
	Prolapse procedures
	Gender dysphoria/gender affirmation surgery
	Interference with sports
	Vaginal atrophy
	Reversal of female genital mutilation
	Body dysmorphia and other psychiatric conditions
	Unsure of the indication for surgery
	Not applicable
20. Have you participated in a clitoral hood reduction during your training program?	
	No
	Uncertain
21. If you have participated in clitoral hood reduction, do you know the indication for the surgery? If you have not seen this procedure, please check not applicable.	Pain
	Sexual dysfunction
	Obstetric-related injuries
	Prolapse procedures
	Gender dysphoria/gender affirmation surgery
	Interference with sports
	Vaginal atrophy
	Reversal of female genital mutilation
	Body dysmorphia and other psychiatric conditions
	Unsure of the indication for surgery
	Not applicable
22. Have you participated in vaginal laser therapy during your training program?	Yes
	No
	Uncertain
23. If you have participated in vaginal laser therapy, do you know the indication for the surgery? If you have not seen this procedure, please check not applicable.	Pain
	Sexual dysfunction
	Obstetric-related injuries
	Prolapse procedures
	Gender dysphoria/gender affirmation surgery
	Interference with sports
	Vaginal atrophy
	Reversal of female genital mutilation
	Body dysmorphia and other psychiatric conditions
	Unsure of the indication for surgery
	Not applicable
24. During a preoperative consultation for cosmetic gynecology procedures, I feel comfortable diagnosing the following conditions: depression, anxiety, and body dysmorphia.	Strongly disagree
	Somewhat disagree
	Neither agree nor disagree
	Somewhat agree
	Strongly agree
25. Based on my training, I will feel comfortable performing cosmetic gynecology procedures independently after graduation.	Strongly disagree
	Somewhat disagree
	Neither agree nor disagree
	Somewhat agree
	Strongly agree
